# [D-Leu^1^]MC-LR and MC-LR: A Small–Large Difference: Significantly Different Effects on *Phaseolus vulgaris* L. (Fabaceae) Growth and Phototropic Response after Single Contact during Imbibition with Each of These Microcystin Variants

**DOI:** 10.3390/toxins12090585

**Published:** 2020-09-11

**Authors:** Luciano Malaissi, Cristian Adrián Vaccarini, Marcelo Paulo Hernández, Marcela Ruscitti, Cecilia Arango, Federico Busquets, Ana María Arambarri, Leda Giannuzzi, Darío Andrinolo, Daniela Sedan

**Affiliations:** 1 Center for Environmental Research (CIM), National Council for Scientific and Technical Research (CONICET), National University of La Plata (UNLP), La Plata 1900, Buenos Aires, Argentina; lucianomalaissi09@hotmail.com (L.M.); cristianvaccarini670@gmail.com (C.A.V.); dandrinolo@yahoo.com (D.A.); 2 Area of Toxicology, School of Exact Sciences, National University of La Plata (UNLP), La Plata 1900, Buenos Aires, Argentina; leda@biol.unlp.edu.ar; 3 Botanical Area, Department of Biological Sciences, School of Agricultural and Forestry Sciences, National University of La Plata (UNLP), La Plata 1900, Buenos Aires, Argentina; marcelo.hernandez@agro.unlp.edu.ar (M.P.H.); fede.bus.1996@gmail.com (F.B.); anaramba@yahoo.com.ar (A.M.A.); 4 Divsion of Vascular Plants, Museum of Natural Sciences of La Plata (UNLP), La Plata 1900, Buenos Aires, Argentina; 5 Institute of Vegetal Physiology (INFIVE), National Council for Scientific and Technical Research (CONICET), National University of La Plata (UNLP), La Plata 1900, Buenos Aires, Argentina; marcelaruscitti@gmail.com (M.R.); mcecilia_arango@hotmail.com.ar (C.A.); 6 Department of Basic and Experimental Sciences, UNNOBA, Junín 6000, Buenos Aires, Argentina; 7 Research Center in Food Cryotechnology (CIDCA), National Council for Scientific and Technical Research (CONICET), La Plata 1900, Buenos Aires, Argentina

**Keywords:** [D-Leu^1^]microcystin-LR, microcystin-LR, imbibition stage, phototropism, phosphatase inhibition

## Abstract

[D-Leu^1^]MC-LR and MC-LR, two microcystins differing in one amino acid, constitute a sanitary and environmental problem owing to their frequent and concomitant presence in water bodies of the Americas and their association with human intoxication during recreational exposure to cyanobacterial bloom. Present in reservoirs used for irrigation as well, they can generate problems in the development of crops such as *Phaseolus vulgaris*, of nutritional and economic interest to the region. Although numerous works address the toxic effects of MC-LR, information on the toxicity of [D-Leu^1^]MC-LR is limited. Our objective was to study the toxic effects of [D-Leu^1^]MC-LR and MC-LR (3.5 µg/ml) on *P. vulgaris* after a single contact at the imbibition stage. Our findings indicate that 10 days post treatment, [D-Leu^1^]MC-LR generates morphological and physiological alterations more pronounced than those caused by MC-LR. In addition to the alterations produced by [D-Leu^1^]MC-LR in the development of seedlings and the structure of the leaves, roots and stems, we also found alterations in leaf stomatal density and conductivity, a longer delay in the phototropic response and a decrease in the maximum curvature angles achieved with respect to that observed for MC-LR. Our findings indicate that these alterations are linked to the greater inhibition of phosphatase activity generated by [D-Leu^1^]MC-LR, rather than to oxidative damage. We observed that 30 days after treatment with MC-LR, plants presented better development and recovery than those treated with [D-Leu^1^]MC-LR. Further studies are required on [D-Leu^1^]MC-LR and MC-LR toxicity and their underlying mechanisms of action.

## 1. Introduction

Cyanobacterial blooms affect the water quality of lakes, ponds and basins around the world. Their presence in these water bodies is favored by eutrophication and climate change [1,2,3]. Cyanobacteria, also known as blue-green algae, are photosynthetic prokaryotes capable in many cases of producing several toxins, known collectively as cyanotoxins. The toxins produced by various species of these microorganisms are grouped into categories according to their chemical characteristics, action mechanisms or the target organs or systems that they preferentially affect [4]. Microcystins (MCs) constitute a cyanotoxin group with high toxicity and large presence in water bodies worldwide. This group is made up of more than 70 structural variants, all of which are cyclic hepatpeptides whose structure has a characteristic amino acid (Adda-(2S,3S,8S,9S)-3-amino-9-methoxy-2,6,8-trimethyl-10-phenyldeca-4,6-dienoic acid) responsible for their toxicity, as well as different L-amino acids at positions 2 and 4 of the molecule. The MCs’ structural variants are formed by modifications in all 7 amino acids, mostly in the L-amino acids at positions 2, 3 (D-erythro-b-methylaspartic acid), 4 or 7 (N-methyldehydroalanine) [5,6]. Microcystin-LR (MC-LR), one of the most frequently found and most toxic MCs, presents the amino acids leucine and arginine at positions 2 and 4, respectively. Other MCs recurrently found in water bodies of the Americas, such as the Río de La Plata river, Salto Grande reservoir (Argentine), Pakowki Lake (Canada) and the Patos Lagoon estuary (Brazil), among others, is [D-Leu^1^]MC-LR, which differs from MC-LR only in the substitution of L-Alanine by D-Leucine at position 1 of the molecule [7,8,9,10,11,12]. Although there is great variability in the amount of MCs produced in cyanobacterial blooms, high levels of MCs have been reported in various parts of the world, probably favored by the environmental conditions and characteristics of the water bodies involved: 28 µg MC-LR/mL (L. Waitawa, New Zealand) [13], 23.7 µg MCs/mL (D. Nhanganzwanein, S. Africa) [14], 13.6 µg MCs/mL (Ingleses beach, Uruguay) and 26.1 µg MCs/mL (Punta Espinillo beach, Uruguay) [15], among others.

Cyanobacterial blooms constitute a major human health problem, impacting on quality of life, and also affecting the organisms inhabiting the ecosystems where they are found. Worldwide, human intoxication through different types of contact with MC-contaminated water have been reported [16,17,18,19,20]. One of the paradigmatic cases of recreational exposure in our region is that of a 20-month-old child who suffered acute liver failure after coming into contact with a dense cyanobacterial bloom on the Carrasco and Malvín beaches (Uruguay) and required a liver transplant. MC levels in the water reached maximum values of 8.2 µg/mL. Both MC-LR and [D-Leu^1^]MC-LR were found in the explanted liver, indicating that these cyanotoxins played a preponderant role in the development of the acute liver failure suffered by the patient [21].

MCs produce their effects through two main mechanisms of action: the inhibition of protein phosphatases (mainly PP1 and PP2A) and the generation of oxidative stress by facilitating the production of reactive oxygen species (ROS) [22,23]. The toxic effects of MCs have been addressed in the literature using various approaches in animal models [24,25,26,27,28,29,30,31,32,33]. Likewise, there are references in the literature to the toxicity that MCs present in plant models, fundamentally using MC-LR. To the best of our knowledge, there are no studies on plant toxicity produced by [D-Leu^1^]MC-LR. Studies on plant physiology have linked the action of PP1 and PP2A inhibitors, like okadic acid, cantaridine and endothall, to alterations in key cellular processes, such as the regulation of genes involved in starch storage, activation of enzymes linked to fixation of CO_2_, activation of sucrose-induced genes, phototropic response and structural alterations in roots [34,35,36,37,38]. In studies addressing the effects of MCs in plant models, the studied routes of exposure have been mainly via irrigation or spraying of the leaves with cyanotoxic blooms or with MC-LR-contaminated water. The authors describe leaf necrosis, growth inhibition, photosynthesis and plant development alterations. These effects are linked not only to inhibitory effects on protein phosphatases, but also to the generation of oxidative stress, resulting in alterations in peroxidase (POD) and superoxide dismutase (SOD) activity, two enzymes that have an important role in the cellular antioxidant system [39,40,41,42,43,44]. However, information on the toxic effects of [D-Leu^1^]MC-LR is scarce in general [7,45]. It has been suggested that [D-Leu^1^]MC-LR could play an ecophysiological role as an antioxidant protector in cyanobacteria [46], or that it could be a possible candidate for the development of an antimycobacterial agent in the treatment of tuberculosis [47]. In a previous study [45], we reported that [D-Leu^1^]MC-LR is able to generate higher inhibition of total protein phosphatase activity in an in vitro test made in mice liver homogenates and in root homogenates than that generated by MC-LR.

The common bean (*Phaseolus vulgaris* L.) is one of the staple native food crops in the Americas and is thus of economic interest to the region; Brazil is the main producer. The crop has low productivity mainly due to the marginality of the lands dedicated to its cultivation, the low input of fertilizers and management techniques, and phytosanitary problems [48]. Given that water from cyanobacteria-contaminated water bodies may be used for irrigation, the effects of cyanotoxins could further affect the productivity of these crops.

In view of the above, the main objective of this work was to evaluate the toxic effects of [D-Leu^1^]MC-LR and MC-LR, two MCs frequently present in our region, on *Phaseolus vulgaris* development after a single contact with each of them during the imbibition stage.

## 2. Results

### 2.1. Macroscopic Alterations

**Figure 1:**
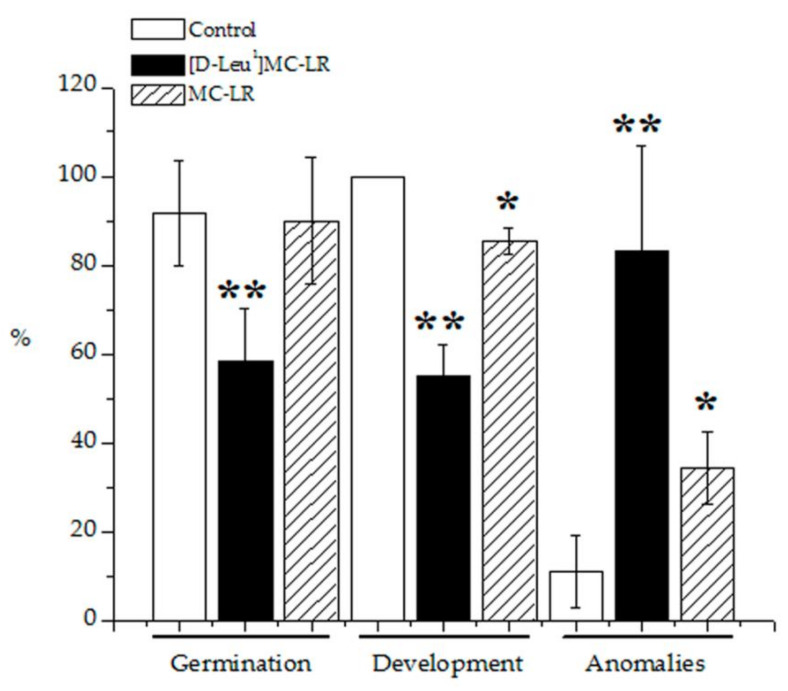
Macroscopic alterations observed at 10 days post imbibition in terms of % of seeds germinated, plants developed and plants with anomalies for the control (white bar), 3.5 µg [D-Leu^1^]MC-LR/mL- (black bar) and 3.5 µg MC-LR/mL- (dashed bar) treated groups. The results are expressed as the mean ± SD (*n* = 20). (*) Indicates significant differences with respect to control values and (**) indicates significant differences between the MCs congeners at the same concentration, according to the ANOVA test of two independent populations (*p* < 0.05).

The germination rate of 3.5 µg [D-Leu^1^]MC-LR/mL-treated seeds was observed 10 days post imbibition to be 58%, which is 37% lower than in the control group (92% germination). Seeds treated with 3.5 µg MC-LR/mL showed no significant differences in germination rate (90%) with respect to the control group (Figure 1). Only 55% of the germinated seeds treated with [D-Leu^1^]MC-LR developed into seedlings compared with 85% for seeds treated with MC-LR, both values significantly lower than for the control group (100%) (Figure 1). Furthermore, the plants developed after treatment with [D-Leu^1^]MC-LR or MC-LR presented alterations at the macroscopic level (stems, leaves and roots), 83% of plants presenting some anomaly in the case of [D-Leu^1^]MC-LR treatment and 34% in the case of MC-LR treatment, both significantly higher than the slight alterations (11%) observed for the control group due to biological variability (Figure 1).

**Figure 2:**
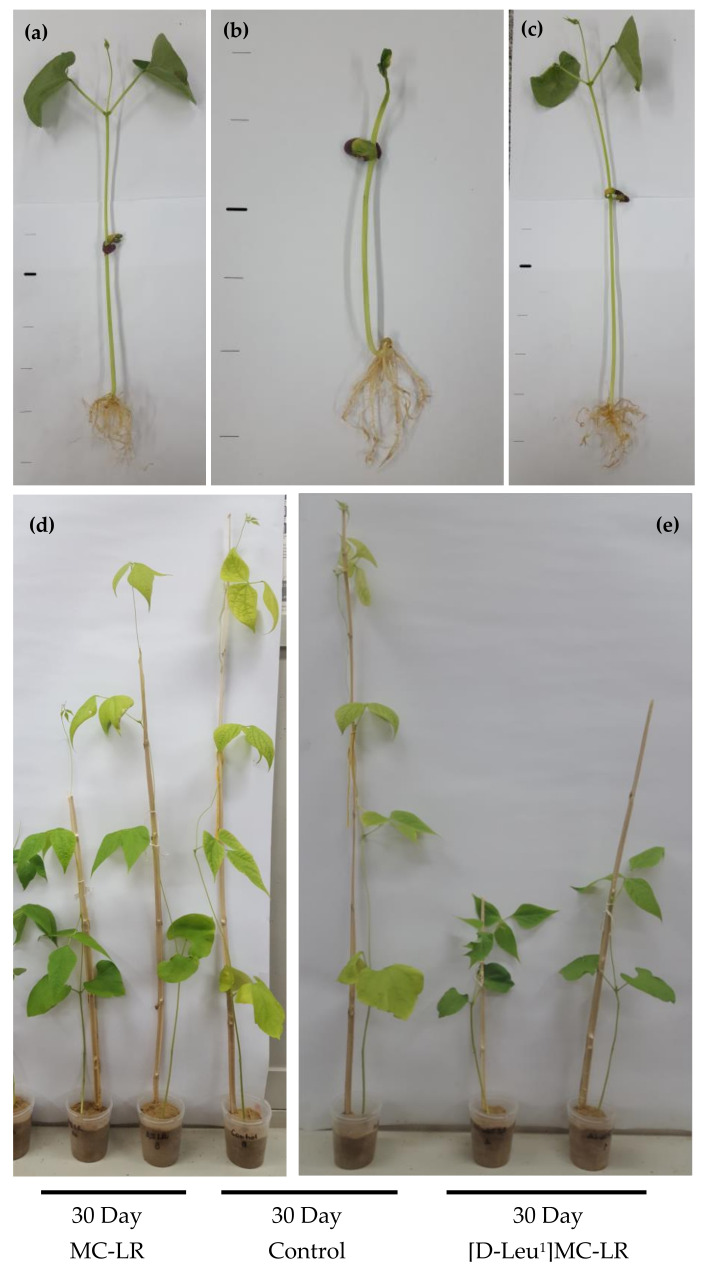
Representative images of developed plants after 10 days ((**a**) control, (**b**) 3.5 µg [D-Leu^1^]MC-LR/mL and (**c**) 3.5 µg MC-LR/mL) and 30 days (**d**,**e**) toxin treatment during the imbibition stage.

**Figure 3:**
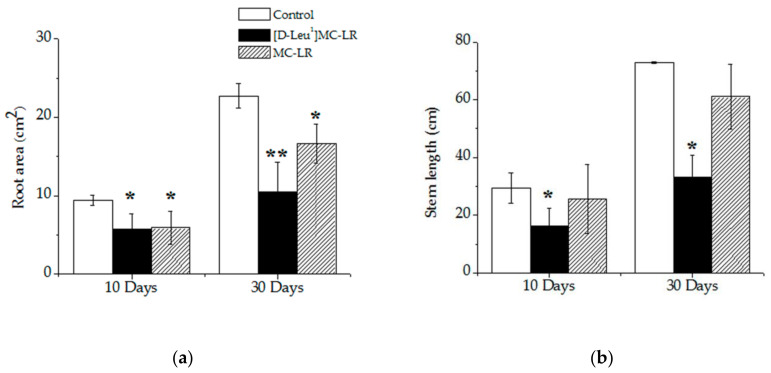
Root area (**a**) and stem length (**b**) of the control (white bar), 3.5 µg [D-Leu^1^]MC-LR/mL- (black bar) and 3.5 µg MC-LR/mL- (dashed bar) treated groups, 10 and 30 days after the imbibition stage. The results are expressed as the mean ± SD (*n* = 20). (*) Indicates significant differences with respect to control values and (**) indicates significant differences between the MCs congeners at the same concentration, according to the ANOVA test of two independent populations (*p* < 0.05).

At 10 days after imbibition, the [D-Leu^1^]MC-LR-treated plants showed shorter stem lengths (Figure 3b), an alteration in the size, color and shape of the leaves (Figure 2b) and a smaller root area with respect to the control (Figure 3a). In MC-LR-treated plants, however, the stem length was similar to the control plants (Figure 3b), the shape of leaves showed slight alterations (Figure 2c) and the root area was smaller than in the controls (Figure 3a). The alterations observed 10 days after contact with the toxins were maintained for 30 days after the imbibition stage (Figure 2d,e), with a shorter stem length and smaller root area observed in the [D-Leu^1^]MC-LR-treated seedlings compared both to the control and to those treated with the same concentration of MC-LR (Figure 3).

**Figure 4:**
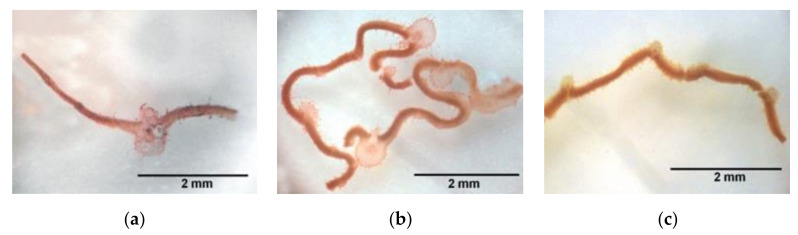
Representative slices of Safranin-stained leaves from the control (**a**), 3.5 µg [D-Leu^1^]MC-LR/mL- (**b**) and 3.5 µg MC-LR/mL- (**c**) treated groups, 10 days after the imbibition stage. Scale lines: 2 mm.

The leaves of the [D-Leu^1^]MC-LR-treated seedlings showed areas of chlorosis of different extension and one of the most distinguishable alterations was the greater undulation observed at the leaf edges (Figure 4b) and the loss of the traditional heart shape of *P. vulgaris* leaves. These alterations in leaf shape were observed 10 days after the imbibition stage and up to 30 days after treatment. The leaves of the seedlings treated with MC-LR presented similar alterations to those produced by [D-Leu^1^]MC-LR, although to a lesser extent (Figure 4c).

We also studied [D-Leu^1^]MC-LR- or MC-LR-treated seeds that had started the germination process but failed to develop a seedling. In these cases, we observed that the main roots usually appeared atrophied and necrotic areas were present in cotyledons treated with [D-Leu^1^]MC-LR (Figure S1).

### 2.2. Microscopic Alterations

**Figure 5:**
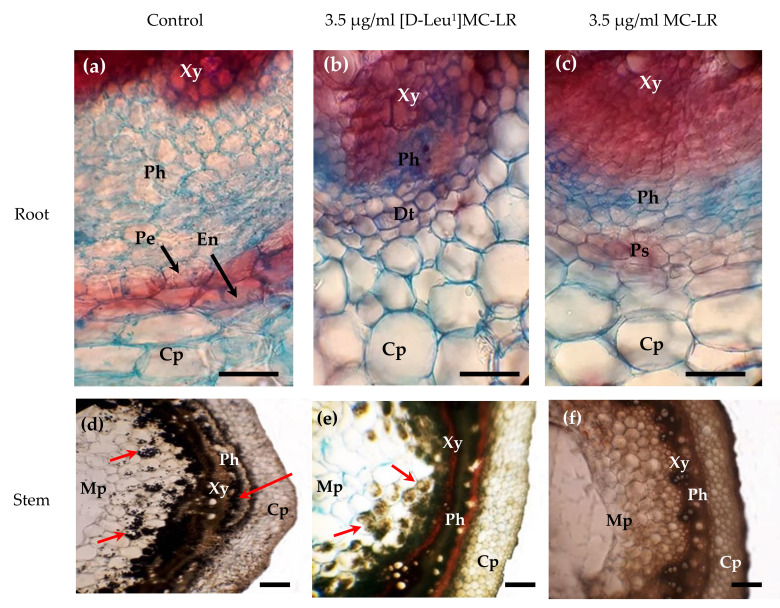
Representative slices of roots (Alcian blue/Safranin stained) and stems (“O” red oil/ IKI stained) from the control (**a**,**d**), 3.5 µg [D-Leu^1^]MC-LR/mL- (**b**,**e**,) and 3.5 µg MC-LR/mL- (**c**,**f**) treated groups, 10 days after the imbibition stage. Xy: Xylem; Ph: Phloem; Pe: Pericycle; En: Endodermis; Cp: Cortical parenchyma; Dt: Disorganized tissue; Ps: Parenchymal sheath; Mp: Medullary parenchyma. Scale lines: 100 µm.

In histological sections of roots treated with 3.5 µg [D-Leu^1^]MC-LR/mL, it was observed that tissue organization had been lost, thus making it impossible to clearly distinguish the pericycle and the endodermis in the disorganized tissue (Figure 5b). A similar situation was observed in the histological sections of the roots of seedlings treated with 3.5 µg MC-LR/mL (Figure 5c). The observed structure is compatible with a more accelerated development of the conduction tissue in seedlings exposed to toxins with respect to the control group (Figure 5a).

These alterations were found 10 and 30 days after treatment with the toxins. In the case of the [D-Leu^1^]MC-LR- or MC-LR-treated seeds that had started the germination process but failed to develop seedlings, we observed secondary roots emerging from the central vascular cylinder, an indication that the vascular tissues develop earlier in the specimens treated with MCs than in the control group (Figure S2). Similarly, in the MC-LR-treated seeds, the presence of roots was observed in the hypocotyl and in the cross section there were raised or “in column” stomata in the epidermis, indicating attempts to develop seedlings under adverse conditions (Figure S3).

Differences were also observed at the microscopic level in the stem, in particular in terms of starch storage. In the case of the control group, the presence of starch granules was observed in all the stem tissues (cortical parenchyma, phloem, xylem and medullary parenchyma) (Figure 5d). However, in seedlings treated with [D-Leu^1^]MC-LR (Figure 5e), starch was only observed in the medullary parenchyma, and in the case of the MC-LR treatment (Figure 5f), starch granules were not observed in any of the stem tissues.

**Figure 6:**
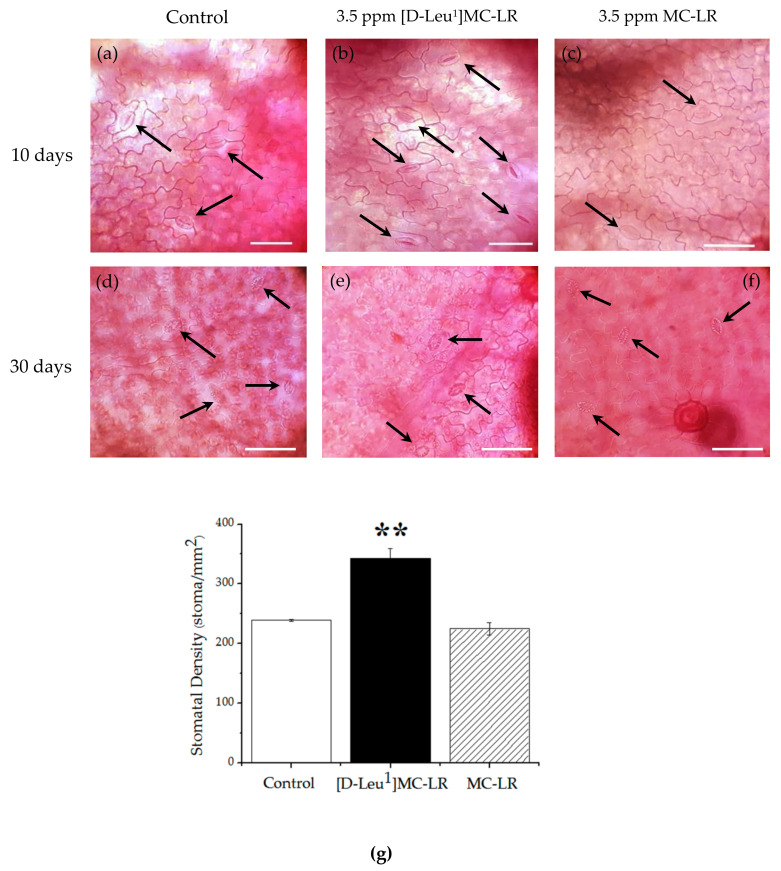
Representative Safranin-stained slices of the abaxial side of leaves of the control group ((**a**) 10 days, (**d**) 30 days), groups treated with 3.5 µg/mL [D-Leu^1^]MC-LR ((**b**) 10 days, (**e**) 30 days) and 3.5 µg/mL MC-LR ((**c**) 10 days, (**f**) 30 days) (scale 100µm). Black arrows indicate stoma. Stomatal density determined as stoma/mm^2^ (**g**) in the control (white bar), 3.5 µg/mL [D-Leu^1^]MC-LR (black bar) and 3.5 µg/mL MC-LR (dashed bar) 10 days post imbibition. The results are expressed as the mean ± SD (*n* = 20). (*) Indicates significant differences with respect to control values and (**) indicates significant differences between the MCs congeners at the same concentration, according to the ANOVA test of two independent populations (*p* < 0.05).

At 10 days post imbibition, an increase in the number of stomata on the abaxial side of the leaves of plants exposed to [D-Leu^1^]MC-LR (Figure 6b) was observed with respect to the control group (Figure 6a). The MC-LR-treated plants (Figure 6c) showed no differences with respect to the controls. Stomatal density was 343 ± 16 stoma/mm^2^ for the [D-Leu^1^]MC-LR treatment group, 225 ± 10 stoma/mm^2^ for the MC-LR treatment group and 239 ± 2 stoma/mm^2^ for the control group (Figure 6g). However, at 30 days after exposure, the groups treated with [D-Leu^1^]MC-LR (Figure 6e) or MC-LR (Figure 6f) showed no significant difference in the number of stomata present on the abaxial side of the leaves with respect to the control group (Figure 6d).

### 2.3. Biochemical Parameters

#### 2.3.1. Pigments

**Figure 7:**
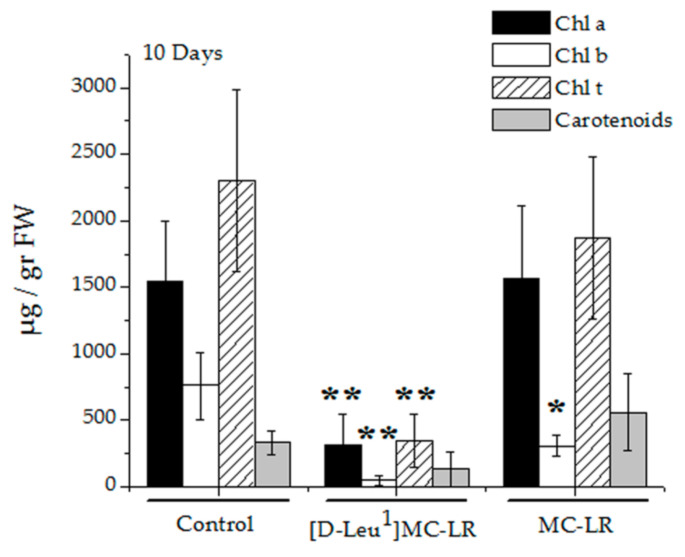
Chlorophyll a (black bar), chlorophyll b (white bar), total chlorophyll (dashed bar) and carotenoids (grey bar) levels in leaves from the control (*n* = 10), 3.5 µg [D-Leu^1^]MC-LR/mL- (*n* = 4) and 3.5 µg MC-LR/mL- (*n* = 6) treated plants 10 days after the imbibition stage. The results are expressed as the mean ± SD. (*) Indicates significant differences with respect to the control values and (**) indicates significant differences between the MCs congeners at the same concentration, according to the ANOVA test of two independent populations (*p* < 0.05).

Treatment with [D-Leu^1^]MC-LR affected chlorophyll a, b and the total levels in leaves 10 days after contact with the toxin during the imbibition stage; the decrease was observed not only with respect to the control but also compared to MC-LR treatment (Figure 7). Leaves of plants treated with MC-LR only showed a decrease in the level of chlorophyll b with respect to the control. However, the carotenoid levels were not significantly altered in any of the microcystin treatments compared to the control group (Figure 7).

#### 2.3.2. Phosphatase Activity

**Figure 8:**
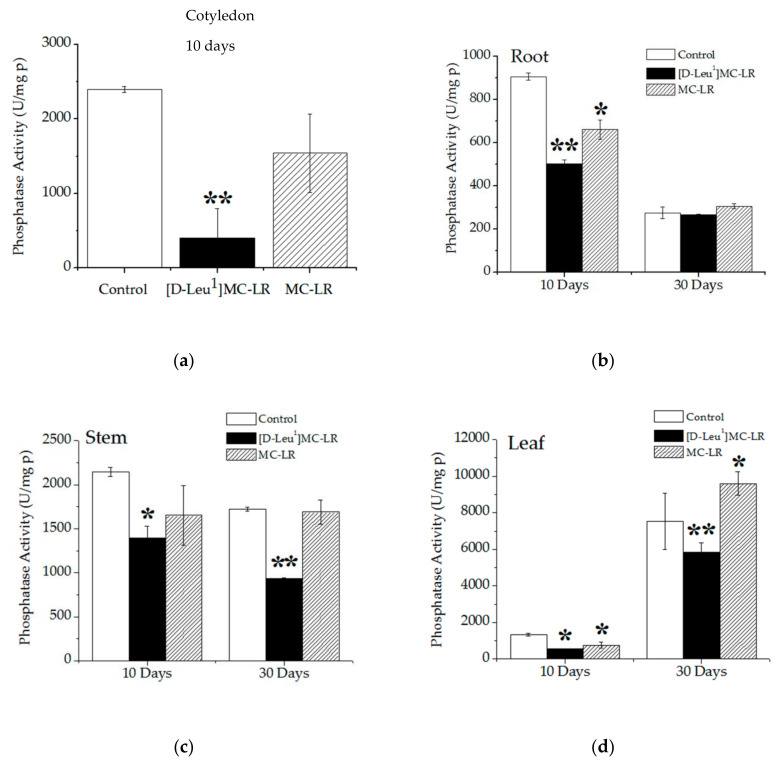
Phosphatase activity in the cotyledon (**a**) 10 days post treatment, root (**b**), stem (**c**) and leaf (**d**) (10 and 30 days post treatment) in the control (white bar), 3.5 µg/mL [D-Leu^1^]MC-LR- (black bar) and 3.5 µg/mL MC-LR- (dashed bar) treated seedlings. The results are expressed as the mean ± SD (*n* = 20). (*) Indicates significant differences with respect to control values and (**) indicates significant differences between the MCs congeners at the same concentration, according to the ANOVA test of two independent populations (*p* < 0.05).

Ten days after the imbibition stage, cotyledons and roots of the 3.5 µg [D-Leu^1^]MC-LR/mL- treated group showed a decrease in total phosphatase activity with respect to the control and 3.5 µg MC-LR/mL-treated seedlings (Figure 8a,b). A decrease in total phosphatase activity was also observed in the stem and leaves of plants treated with [D-Leu^1^]MC-LR compared to the control plants (Figure 8c,d). However, treatment with MC-LR only generated a decrease in total phosphatase activity in roots and leaves 10 days after contact with the toxin.

Total phosphatase activity continued to decrease in the stems and leaves of the [D-Leu^1^]MC-LR-treated seedlings 30 days after toxin treatment, while an increase in total phosphatase activity was observed in the leaves of plants exposed to MC-LR (Figure 8c,d).

#### 2.3.3. Lipid Peroxidation

**Figure 9:**
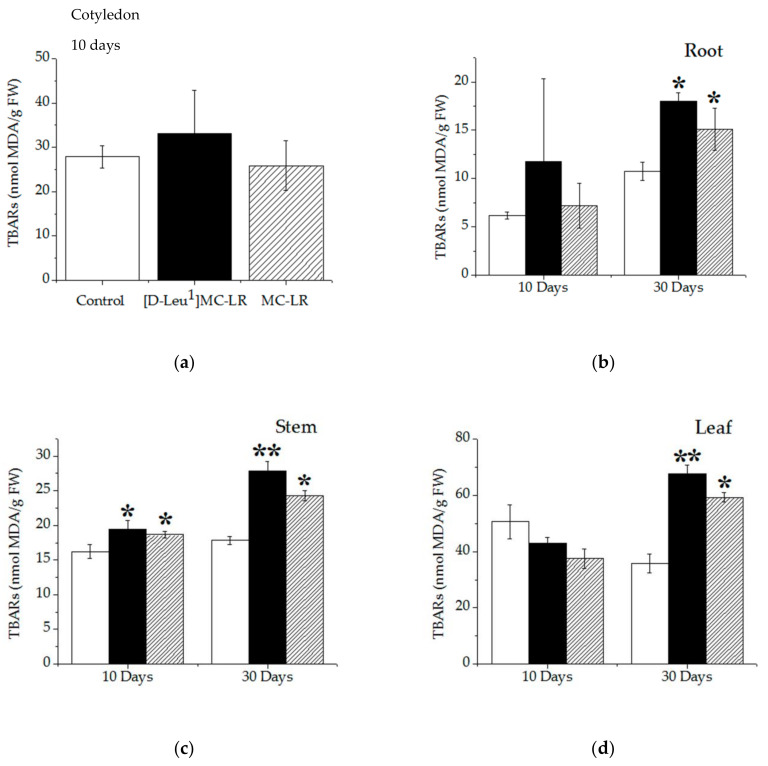
Lipid peroxidation levels determined as thiobarbituric acid reactive substances (TBARS) (nmol MDA/g FW) in the cotyledon (**a**) 10 days post treatment, root (**b**), stem (**c**) and leaf (**d**) (10 and 30 days post treatment) of the control (white bar), 3.5 µg/mL [D-Leu^1^]MC-LR- (black bar) and 3.5 µg/mL MC-LR- (dashed bar) treated seedlings. The results are expressed as the mean ± SD (*n* = 20). (*) Indicates significant differences with respect to control values and (**) indicates significant differences between the MCs congeners at the same concentration, according to the ANOVA test of two independent populations (*p* < 0.05).

Thiobarbituric acid reactive substances (TBARS), measured as malondialdehyde (MDA), were determined in plant tissues, indicating lipid peroxidation. Ten days after toxin contact, a slight increase in the TBARS levels was observed only in the stems of the [D-Leu^1^]MC-LR- and MC-LR -treated groups (Figure 9c). In the cotyledon, roots and leaves of the toxin-treated plants, the TBARS levels were no different from the control group (Figure 9a,b,d).

Increases in the TBARS levels were observed 30 days post imbibition in the roots, stems and leaves of plants treated with both toxins, compared to the controls (Figure 9b–d). The TBARS levels in the stems and leaves of the 3.5 µg [D-Leu^1^]MC-LR/mL-treated plants were significantly higher than in the controls and in those treated with 3.5 µg MC-LR/mL.

### 2.4. Physiological Alteration

#### 2.4.1. Stomatal Conductivity

**Figure 10:**
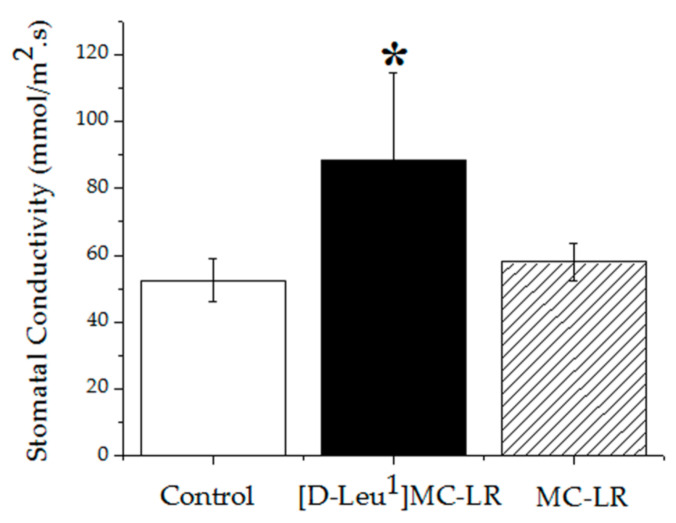
Stomatal conductivity (mmol/m^2^.s) in leaves of the control (white bar), 3.5 µg [D-Leu^1^]MC-LR/mL- (black bar) and 3.5 µg MC-LR/mL- (dashed bar) treated groups 10 days after treatment. (*) Indicates significant differences with respect to the control values and (**) indicates significant differences between the MCs congeners at the same concentration, according to the ANOVA test of two independent populations (*p* < 0.05).

At 10 days post imbibition, an increase in stomatal conductivity was observed in the leaves of the [D-Leu^1^]MC-LR-exposed plants compared to the control group. The stomatal conductivity values of the MC-LR-treated plants showed no differences with respect to the control group (Figure 10). This result correlates with the increase in stomatal density observed 10 days post treatment with [D-Leu^1^]MC-LR.

At 30 days post imbibition there were no significant differences in the values of stomatal conductivity between the groups treated with toxins and the control group.

#### 2.4.2. Phototropic Response Alterations

**Figure 11:**
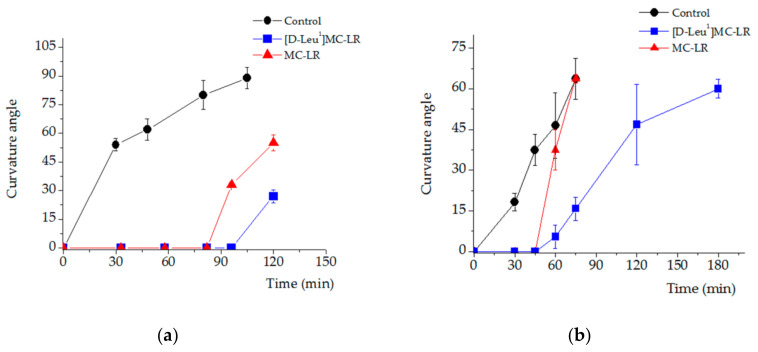
Phototropic response. Curvature angle observed in response to a point light source as a function of time for the control (black), 3.5 µg [D-Leu^1^]MC-LR/mL- (blue) and 3.5 µg MC-LR/mL- (red) treated- seedlings; 10 days (**a**) and 30 days (**b**) post imbibition. The results are expressed as the mean ± SD (*n* = 20). (*) Indicates significant differences with respect to the control values and (**) indicates significant differences between MCs congeners at the same concentration, according to the ANOVA test of two independent populations (*p* < 0.05).

Our results indicate a delay in the phototropic response of seedlings treated with 3.5 µg [D-Leu^1^]MC-LR/mL and 3.5 µg MC-LR/mL, 10 days after imbibition, compared to the control group. After 30 min of exposure to a point light source, the control seedlings showed a curvature angle of 54 ± 3° whereas seedlings treated with [D-Leu^1^]MC-LR or MC-LR showed no response (Figure 11a). The [DLeu^1^]MC-LR-treated seedlings showed the longest delay in phototropic response, reaching a curvature angle of 27 ± 3° after 120 min. In the case of MC-LR-treated plants the curvature angle was 33 ± 2° at 96 min. The lowest maximum curvature angle reached during the study of phototropic effects was in treated plants. Thus, while the control presented an angle of 89 ± 6° at 105 min, the seedlings treated with MC-LR showed an angle of 55 ± 4° at 120 min. The lowest curvature angle recorded during the study was that observed in seedlings treated with [D-Leu^1^]MC-LR, which presented a curvature angle of 27 ± 3° at 120 min.

Evaluation of the phototropic response in seedlings 30 days post exposure showed a delayed response: seedlings exposed to MC-LR and [D-Leu^1^]MC-LR began to curl after 45 min of exposure to a spot light whereas seedlings in the control group began to respond within a few minutes of light exposure (Figure 11b). Thus, the curvature angles reached 60 min after unilateral light exposure were 46 ± 12° for the control group, 37 ± 7° for those treated with MC-LR and 5 ± 4° in the case of seedlings treated with [D-Leu^1^]MC-LR. After 75 min of unilateral light exposure, the seedlings of the control group (64 ± 7°) and the group treated with MC-LR (64 ± 2°) reached similar values of curvature angles. However, seedlings treated with [D-Leu^1^]MC-LR were only able to reach a curvature angle comparable to that previously described (60 ± 4°) after 120 min of unilateral light exposure.

## 3. Discussion

The toxic effects of MC-LR on different plant species have been evaluated in several studies, in which exposure was usually carried out by prolonged irrigation or surface treatment on the leaves [40,42,44,49,50,51]. However, the effects produced on plants by [D-Leu^1^]MC-LR, a MC-LR congener commonly present in water bodies of the Americas, have not been studied. The findings reported in the literature indicate that the resulting toxic effects are not only concentration- and/or MCs congener-dependent, but are also influenced by the sensitivity of the plant species in question and by whether the treatments involved crude extracts or purified toxins. Abe et al. [40] found leaf necrosis and a reduction in the rate of photosynthesis in *P. vulgaris* by repeatedly dipping leaves into an MC-LR solution. Studies carried out on the prolonged exposure of *L. esculentum* to extracts of cyanobacteria, containing MCs levels between 2.22 and 22.24 µg/mL, showed a reduction in germination, inhibition of growth and productivity, alterations in photosystem II activity, tissue necrosis of leaves and promotion of oxidative stress [51]. Mc Elhiney et al. [42] observed alterations in root development of germinated *P. vulgaris* seeds after exposure to MC-LR in culture media between 3 and 18 days. This root alteration is correlated with a decrease in the uptake of culture media. These authors also observed inhibition in the growth and chlorophyll content of *Solanum tuberosum* L. and *Synapis alba* L. seedlings. Furthermore, *Medicago sativa* seedlings exposed to 5 µg MC-LR/L for seven days exhibited inhibition of germination and root growth and oxidative damage (lipid peroxidation and elevated reactive oxygen detoxifying enzymes) [49]. Similarly, *Triticum aestivum* irrigated with MCs or crude cyanobacteria extract every 3 days for 6 weeks showed morphological alteration in the roots and shoots, together with inhibition of photosynthesis and the elevation of antioxidative-response enzymes [50].

In the present study, we carried out a comparative analysis of the toxic effects caused by a single contact during the imbibition stage of two MCs, MC-LR and [D-Leu^1^]MC-LR, which only differ in one amino acid. These two MCs are usually present concomitantly in water bodies of our region and therefore constitute a sanitary and environmental problem. This is the first study to evaluate the exposure route via a single contact during seed imbibition, a fundamental stage in seed germination, when the biochemical and physiological processes leading to development of the seedling are activated. This exposure route is of considerable importance from the toxicological and economic points of view given its potential impact on crop production. Although the toxin concentration used in the treatments may be high compared to those commonly found in the environment, it was selected on the basis of previous work carried out by our research group [45] to reflect extreme situations in which eutrophic water from reservoirs with highly toxic blooms is used for incidental crop irrigation.

Our findings indicate [D-Leu^1^]MC-LR to have greater toxic potency than MC-LR, as evidenced by a greater decrease in germination and seedling development (37% and 45%, respectively) and a higher proportion of anomalies (83%) after treatment with [D-Leu^1^]MC-LR. Saqrane et al. [44] observed a decrease in the germination of several treated seeds after 4 days of exposure to crude extracts at concentrations of 2.9 µg MC-LReq/mL or higher, *Lens esculenta* being the most resistant variety and *Pisum sativum* the most sensitive. In our case, after a single contact during the imbibition stage with 1 mL of 3.5 µg/mL [D-Leu^1^]MC-LR, we observed similar results to those found by the latter authors in *P. sativum* after 4 days of contact with 10 mL of 1.69 µg MC-LReq/mL.

Similarly, and in agreement with the results reported for prolonged exposures [49,50], we observed alterations in root development as indicated by a decrease in root area 10 days post imbibition. Again, this alteration was more evident in the specimens exposed to [D-Leu^1^]MC-LR; after 30 days in the absence of contact with the toxin there was no sign of damage recovery, whereas in the case of MC-LR there was a reversal towards control values. Stem length was not significantly affected after a single contact with MC-LR, whereas in the case of [D-Leu^1^]MC-LR the stems were significantly shorter both 10 and 30 days post treatment. More severe undulation of leaf edges was observed for the [D-Leu^1^]MC-LR-treated group.

At the histological level we observed root alterations similar to those reported by other authors in *P. sativum* after 8 days of exposure to 11.6 µg MC-LReq/mL [44], mainly in terms of disorganized tissue that made it difficult to differentiate between the pericycle and the endodermis. In the hypocotyl of MC-LR-treated seeds that had initiated but failed to complete the germination process we observed roots and raised or “in column” stomata, indicating attempted development under the adverse conditions of exposure to MC-LR. However, we did not observe this in the case of [D-Leu^1^]MC-LR treatment, instead finding atrophied primary roots and necrotic areas in cotyledons, indicating more severe damage. This lack of attempted seed development is in line with the higher toxic potency of the latter toxin with respect to MC-LR.

Our findings indicate that both toxins affected the starch granule deposits. It has been proposed that starch deposits in plants act not only as carbon reserves, but also as an energy buffer to mobilize and reuse energy supplies during development [52]. Treatment with [D-Leu^1^]MC-LR can affect not only the starch deposit mechanisms, but also the mechanisms for reutilizing the starch during development. The poor growth in the [D-Leu^1^]MC-LR-treated plants together with the alteration in starch deposits observed, indicates interference with the starch deposit mechanism, also preventing the subsequent reuse of starch during plant development. In the case of treatment with MC-LR, the starch deposit mechanism is less affected and starch mobilization and reuse appears to remain viable, such that these plants are better able to recover and develop than those treated with [D-Leu^1^]MC-LR.

Numerous studies describe alterations in chlorophyll and photosystem II after exposure to MCs for several days [40,42,50,51]. We observed alterations in chlorophyll after a single contact with the toxins, these being more marked after [D-Leu^1^]MC-LR than MC-LR exposure.

Taken together, the above findings reinforce the higher toxic potency of [D-Leu^1^]MC-LR than MC-LR.

In previous publications [49,51,53], increases in lipid peroxidation levels, as well as in various components of the cellular antioxidant system (catalase, superoxide dismutase, peroxidase, glutathione reductase, glutathione-S-transferase, α- and β-tocopherol) have been described in seedlings exposed for several days to crude extracts containing MCs. In the current study, 10 days after contact with the studied toxins, we observed elevated TBARS levels only in stems, one of the most affected tissues by this treatment. However, 30 days post treatment, the levels of the TBARS were higher in all plant tissues studied and, in the case of leaves and stems, significantly higher for [D-Leu^1^]MC-LR than for MC-LR exposure. Given that we used a neutral substrate (sand) for crop development, it is possible that the oxidative stress observed 30 days after treatment derived in part from the combined action of the MCs and the environmental stress caused by nutrient depletion typical of this growth stage under these conditions.

It is recognized that, in addition to the induction of oxidative stress, the inhibition of protein phosphatase activity (PP1 and PP2A) constitutes one of the main mechanisms of action of MCs. Our findings indicate that at 10 days post imbibition, [D-Leu^1^]MC-LR severely inhibited total phosphatase activity in tissues such as cotyledons, stems, roots and leaves, and caused lower total phosphatase activity values in the cotyledons and roots with respect to MC-LR. This inhibition of total phosphatase activity in tissues such as cotyledons, where no increase in TBARS was observed, could explain the strong inhibitory effects on the germination and development of *P. vulgaris* seeds exposed to a single contact with [D-Leu^1^]MC-LR, since phosphatase protein activity (mainly PP2A) is strongly involved in regulating several physiological processes that occur in germination and plant development [54,55]. Takemiya et al. [56] have proposed that stomatal function is regulated by pathways in which protein phosphatases are involved, specifically PP1, intervening in the dynamics of stomatal opening to certain light stimuli. In our study we observed an increase in the stomatal conductivity secondary to an increase in stomatal density, 10 days after treatment with [D-Leu^1^]MC-LR. Therefore, this physiological alteration could constitute an adaptive response to the inhibition of phosphatase activity induced by the toxin. We carried out a determination of the total phosphatase activity with a non-specific substrate (pNpp); therefore, and as we observed in previous studies [32,45], we must consider the possibility that [D-Leu^1^]MC-LR has the ability to inhibit other phosphatases that may be involved in physiological processes. In seedlings developed 30 days post exposure to [D-Leu^1^]MC-LR, total phosphatase activity continued to be inhibited in stems and leaves whereas in the MC-LR treatment, the phosphatase activity values were similar to those of the control group, except in leaves, where we found an increase in the total phosphate activity, probably indicating an attempt at recovery. These parameters, together with macroscopic observations, indicate the better recovery of seedlings exposed to MC-LR than those exposed to [D-Leu^1^]MC-LR.

To the best of our knowledge, this is the first study showing the effects of MC-LR and [D-Leu^1^]MC-LR on phototropism in plants, demonstrating a significant change in the physiological process of plants’ adaptation to the environment. The effect is greater in plants exposed to [D-Leu^1^]MC-LR, since they present a longer delay in response and reach a lower curvature angle. These alterations are manifested even in seedlings that continued development for 30 days (toxin-free watering) after initial contact with the toxin. There are studies that link alterations in auxins with variations in phototropic response [38]. The inhibition of protein phosphatases could cause the synthesis or action of auxins to be interrupted or affected, which would impact on germination and seedling development. Furthermore, Padmale and Liscum [37] showed alteration at the phototropism level by protein phosphatase inhibitors, such as okadaic acid; the same could occur with MC-LR and [D-Leu^1^]MC-LR, since they inhibit the same protein phosphatases as okadaic acid [4]. Working with PP2A loss-of-function *Arabidiopsis thaliana* mutants or with the inhibition of PP2A by cantharidine, Rashotte et al. [57] and Michniewicz et al. [58] determined that the inhibition of PP2A activity caused alterations in root development and in gravity response. Their findings indicate that phosphatase inhibition produces a basal-to-apical PIN polarity shift in embryos and roots that generates an altered auxin distribution, leading in turn to poor development of the main root, favoring secondary roots and a delayed gravity response with respect to the control groups. In this sense, and considering the inhibition of the protein phosphatase activity observed in our study, it is possible that alteration in PIN protein polarity and the consequent variation in auxin transport may be the mechanism underlying the delay in phototropic response and the alterations observed in the development of *P. vulgaris* treated with [D-Leu^1^]MC-LR and MC-LR. However, it is possible that one of the differences that generates the higher toxic potency of [D-Leu^1^]MC-LR compared to MC-LR is the ability of [D-Leu^1^]MC-LR to inhibit other phosphatases or esterases in addition to PP1 and PP2A. This could explain the greater delay in the response observed during the phototropism test, as well as the anomalies detected in the other parameters analyzed.

In plants, it has been reported that alterations in the development of plant organs and tissues are closely linked to chromatin organization, histone phosphorylation and PIN protein, all key regulators in a large number of processes, such as axis development in embryogenesis, organogenesis, root meristem organization and tropisms [58,59]. Likewise, it has been proposed that the inhibition of phosphatase activity (mainly PP2A and PP1, although not exclusively) generates strong alterations in the aforementioned processes, leading to the damage observed at the macro and microscopic levels. Our findings, taken together, indicate that the damage observed after a single contact exposure to [D-Leu^1^]MC-LR and MC-LR derives mainly from the inhibition of phosphatase activity that these toxins produce, rather than from alteration of the oxidative state in *P. vulgaris*.

## 4. Conclusions

In summary, our results indicate that a single contact with MC-LR or [D-Leu^1^]MC-LR during the imbibition stage produces alterations in the development and physiological processes of *P. vulgaris* that are fundamentally linked to the inhibition of phosphatase activities. Although the concentration used in this study is relatively high (3.5 µg/ml), it resembles extreme conditions under which water sources with cyanobacterial blooms are used incidentally for irrigation, in particular when pre-treatment of the water for clarification purposes releases large amounts of toxins into the water.

It is evident that even though [D-Leu^1^]MC-LR and MC-LR only differ in one amino acid in their molecule, they have different toxic potencies. The more pronounced toxic effects in the case of [D-Leu^1^]MC-LR makes it more difficult for seedlings to recover from exposure to this toxin compared to that observed in the case of MC-LR, once contact with the toxin has ended.

Given the environmental and public health problem generated by the presence of [D-Leu^1^]MC-LR and MC-LR in freshwater bodies of the Americas, leading to the occurrence of human and animal intoxication events, further research into the effects of these toxins in plant and animal models is called for.

## 5. Materials and Methods

### 5.1. [D-Leu^1^]MC-LR and MC-LR Purification

[D-Leu^1^]MC-LR and MC-LR were purified from natural blooms of *Microcystis aeruginosa* collected in the Río de la Plata river, as described in previous studies [30,31,33,60]. Briefly, cells were broken by sonication (Omni-Ruptor 400, 15 min) and the extract treated with chloroform/methanol (50:50; *v*:*v*). The aqueous fraction was concentrated in a rotavapor (Decalab, R-23, Buenos Aires, Argentina). Purification was performed with semi-preparative high-performance liquid chromatography. We used a Shimadzu 20A HPLC apparatus with a degassed module and a diode array detector system set at 238 nm. The preparative column used was a TERMO Hyperprep HS C18 (250 10 mm) and the mobile phase was deionized water (TFA 0.05%) with 35% acetonitrile (TFA 0.05%) run in gradient conditions at 5 mL/min. The peak corresponding to [D-Leu^1^]MC-LR or MC-LR was collected separately and after evaporating the acetonitrile in a rotavapor, was concentrated with a previously activated C18 cartridge. Pure [D-Leu^1^]MC-LR and MC-LR were eluted with a solution of methanol:water (90:10, *v*:*v*) after which the methanol was evaporated in a rotavapor. Final identification and concentration were achieved by comparison with a Sigma Chemical Inc. toxin standard (St. Louis, MO, USA).

### 5.2. [D-Leu^1^]MC-LR and MC-LR Acute Exposure in Phaseolus vulgaris

Three groups each of forty healthy-looking *Phaseolus vulgaris* seeds were employed for the MCs’ acute exposure evaluation. The toxins were prepared freshly by dilution of the stock preparation with sterile MC-free water. One group of seeds was exposed to 3.5 µg/mL of [D-Leu^1^]MC-LR and another to 3.5 µg/mL of MC-LR. We chose *P. vulgaris* for our study since it is a food crop of nutritional and economic interest to the region and is susceptible to irrigation with eutrophic water during the commencement of seed germination. The chosen concentration for the experiments is higher than that usually found in water bodies in the region, though there are cases [53] in which such high levels of toxins have been determined, reflecting extreme conditions of concentrated blooms or the use of pre-treated water to clarify them with a concomitant release of large amounts of toxin into the water. The corresponding control group of seeds was treated with equivalent volumes of sterile MC-free water. Acute toxin exposure was carried out by placing the seeds in contact with [D-Leu^1^]MC-LR or MC-LR only during the imbibition stage. Each seed was placed in a test tube with 1 mL of 3.5 µg MC-LR/mL, 3.5 µg [D-Leu^1^]MC-LR/mL solution or sterile MC-free water. The tubes were placed in a temperature chamber (24 ± 1 °C) for 24 h (imbibition stage). Then, each seed was sown in sterile sand, used as a neutral substrate, moistened with sterile MC-free water and maintained in a temperature chamber (24 ± 1 °C), under 14 h-light/dark cycles and 42 μmol photons/m^2^s light intensity for 10 and 30 days to allow their germination and subsequent seedling development. The seeds were irrigated daily with sterile MC-free water and macroscopic observations were carried out throughout the test. Samples were taken 10 (*n* = 20) and 30 (*n* = 20) days after imbibition from each treatment and the control group. In each case, different plant tissues (root, stem, leaves and cotyledons) were cut and separated, weighed and a photographic record of each structure was made for subsequent analysis.

### 5.3. Macroscopic Determinations

#### 5.3.1. Germination, Development and Anomalies Percentages

*P. vulgaris* germination was determined as the percentage of germinated seeds relative to the total seeds for each group. Development was determined as the percentage of seeds that germinated and developed a seedling 10 days post treatment with respect to the total germinated seeds for each group. We also determined the percentage of plants that showed any macroscopic anomaly after 10 days of development without contact with the toxins, with respect to total developed seedlings in each group. In the case of the control group, slight macroscopic anomalies due to biological variability were taken into account in order to establish an adequate base line.

#### 5.3.2. Root Area and Stem Length

ImageJ software (free access software, available on https://imagej.softonic.com) was used to carry out the morphometric analysis. The root area was determined from photographs taken of the roots at the 10 and 30 days post imbibition stage through image processing and the use of reference scales. In the same way we determined the length of the stems.

### 5.4. Histological Analysis

A sub-sample of each tissue was placed in FAA (formalin-acetic acid-alcohol) 70% fixation liquid [61]. For stomatal density analysis, foliar diaphanizations [62] and safranin monochromatic staining were performed.

Longitudinal and cross sections were made of fixed tissues, bleached in 10% sodium hypochlorite (NaOCl) for 5–10 minutes and washed three times with distilled water. For the anatomical study, successive double staining with Alcian blue and safranin was performed [63]. To detect lipophilic substances, an alcoholic solution of “O” red oil was used [64]; starch grains were identified with iodine-potassium iodide (IKI) [65].

In each technique, the material was mounted in glycerin water 1:1 on glass slides; finally, preparations were sealed with nail polish. Histological analysis was carried out using two slides per sample and studying 3 microscopic fields in each one. For the stoma count, anatomical and histochemical analysis, a light microscope (Leitz SM Lux) with a lucid camera was used. A CCD PAL color camera connected to a Gemalux microscope allowed us to digitize the images captured using Hyper Media Center software.

### 5.5. Biochemical Parameters

#### 5.5.1. Homegenates and Leaf Samples

At 10 and 30 days post treatment, one representative leaf of seedlings from the treated and control groups was cut and weighed in order to determine pigment content.

Samples of cotyledons (10 days post imbibition) and roots, stems and leaves (10 and 30 days post imbibition) were placed in an ice-cold buffered solution (20 mM buffer phosphate, pH 7.0) and homogenized by means of a mortar. Cell debris and nuclei were removed by centrifugation at 10,000× *g* (20 min at 1–2 °C) in a Sorvall RC5C Dupont centrifuge (Block Scientific, New York, USA). Supernatants were aliquoted and frozen in hermetic polypropylene vials at −70 °C under an N_2_ atmosphere until use for lipid peroxidation and phosphatase activity measurements.

#### 5.5.2. Pigment Determination

Leaf chlorophyll and carotenoid levels were determined according to the method described by Lichtenthaler et al. [66]. Briefly, each leaf was cut with scissors, placed in a test tube containing methanol and kept at −20 °C in the dark overnight. The cell debris were then separated by centrifugation at 5000 rpm in a Rolco centrifuge and the colorimetric determination made in the supernatant according to the cited method. Absorbance was measured at 665, 652 and 470 nm employing a UV–Vis spectrophotometer (Infinite 200Pro). The chlorophyll a (Chl *_a_*), b (Chl *_b_*), total chlorophyll (Chl *_t_*) and carotenoids levels were calculated using the equation 1–4:

Chl*_a_*= 16.72 × A_665_ − 9.16 × A_652_ (1)

Chl*_b_*= 34.09 × A_652_ − 15.28 × A_665_ (2)

Chl *_t_*= 1.44 × A_665_ − 24.93 × A_652_ (3)

Carotenoids = (1000 × A_470_ − 1.63 × Chl*_a_* − 104.96 × Chl*_b_*)/221 (4)

#### 5.5.3. Lipid Peroxidation

To determine lipid peroxidation, samples from the cotyledons, stem, roots and leaf homogenates were processed for thiobarbituric acid reactive substances (TBARS) and measured as malondialdehyde (MDA) according to the colorimetric method described by Okawa et al. [67]. The samples were analyzed in triplicate.

#### 5.5.4. Protein Phosphatase Activity Assay

The protein phosphatase activity assay was carried out in triplicate on tissue homogenates following the protocol described by Chen et al. [68]. Para-nitrophenyl phosphate (p-NPP), diluted in a 50 mM Tris–HCl buffer containing 0.1 mM EDTA, 5 mM dithiothreitol, 0.2 mM MnCl_2_ and 0.2 mg/mL bovine serum albumin at pH 7.0, was employed as the substrate. One unit (U) was defined as the phosphatase activity that hydrolyzes 1 nmol of para-nitrophenyl phosphate (p-NPP) in 1 min. The assay was carried out at 30 °C during 1 h and after the stop reaction with 0.5 N NaOH solution, the absorbance was determined at 405 nm. The phosphates activity for each toxin treatment was calculated by the change in absorbance and expressed as a percentage of the activity of the control.

### 5.6. Physiological Determination

#### 5.6.1. Stomatal Conductivity

At 10 and 30 days post treatment, before taking samples, stomatal conductivity was determined “in vivo” in plants from the toxin-treated and control groups. The measurements were carried out in culture conditions by placing a porometer (METER GROUP Porometer SC-1) sensor on the leaves of each plant studied. Unless impossible due to the small size of the leaf, each measurement was made in three different sectors of the leaf and an average obtained.

#### 5.6.2. Phototropic Response

Before taking samples, the phototropic response was evaluated “in vivo” 10 and 30 days after the imbibition stage on seedlings of the control and toxin-treated groups. Briefly, seedlings were placed in a box with dark walls to avoid light reflection and with a side window where the point light source was placed. Once the test started, the curvature angle reached by each plant was determined, employing a protractor, as a function of time.

### 5.7. Statistical Analysis

All results were subjected to one-way analysis of variance (ANOVA) with the aid of Systat (version 12.0 for Windows) from SPSS Science (17.0.1, Chicago, IL, USA, 2008), and represent the means ± SD of 20 seeds per group. Differences in the mean value between groups were assessed by a two-tailed Student’s t-test and a *p* < 0.05 was considered statistically significant.

## Supplementary Materials

The following are available online at https://www.mdpi.com/2072-6651/12/9/585/s1, Figure S1: Representative seeds treated with [D-Leu^1^]MC-LR (a,b) or MC-LR (c,d) that started the germination process but failed to develop a seedling. Figure S2: Representative histological sections stained with Alcian Blue/Safranin (40×) from roots of seeds treated with [D-Leu^1^]MC-LR (a) or MC-LR (b) that started germination but failed in seedling development. Figure S3: Representative hypocotyl surface view (a,b) and Alcian Blue/Safranin-stained cross section (c,d) (100×) from MC-LR-treated seeds that started germinating but failed to develop seedlings.
