# Supplementary material for: [D-Leu1]MC-LR and MC-LR: A Small–Large Difference: Significantly Different Effects on Phaseolus vulgaris L. (Fabaceae) Growth and Phototropic Response after Single Contact during Imbibition with Each of These Microcystin Variants
Source: Toxins (Basel). 2020 Sep 11;12(9):585. doi: 10.3390/toxins12090585 (PMC7551030; doi:10.3390/toxins12090585)
Supplement: Supplementary file 1 [file toxins-12-00585-s001.pdf]

Supplementary Materials: [D-Leu¹]MC-LR and MC-LR: A Small-Large Difference: Significantly Different Effects on *Phaseolus vulgaris* L. (Fabaceae) Growth and Phototropic Response After Single Contact During Imbibition with Each of These Microcystin Variants

Luciano Malaissi, Cristian Adrián Vaccarini, Marcelo Paulo Hernández, Marcela Ruscitti, Cecilia Arango, Federico Busquets, Ana María Arambarri, Leda Giannuzzi, Darío Andrinolo and Daniela Sedan

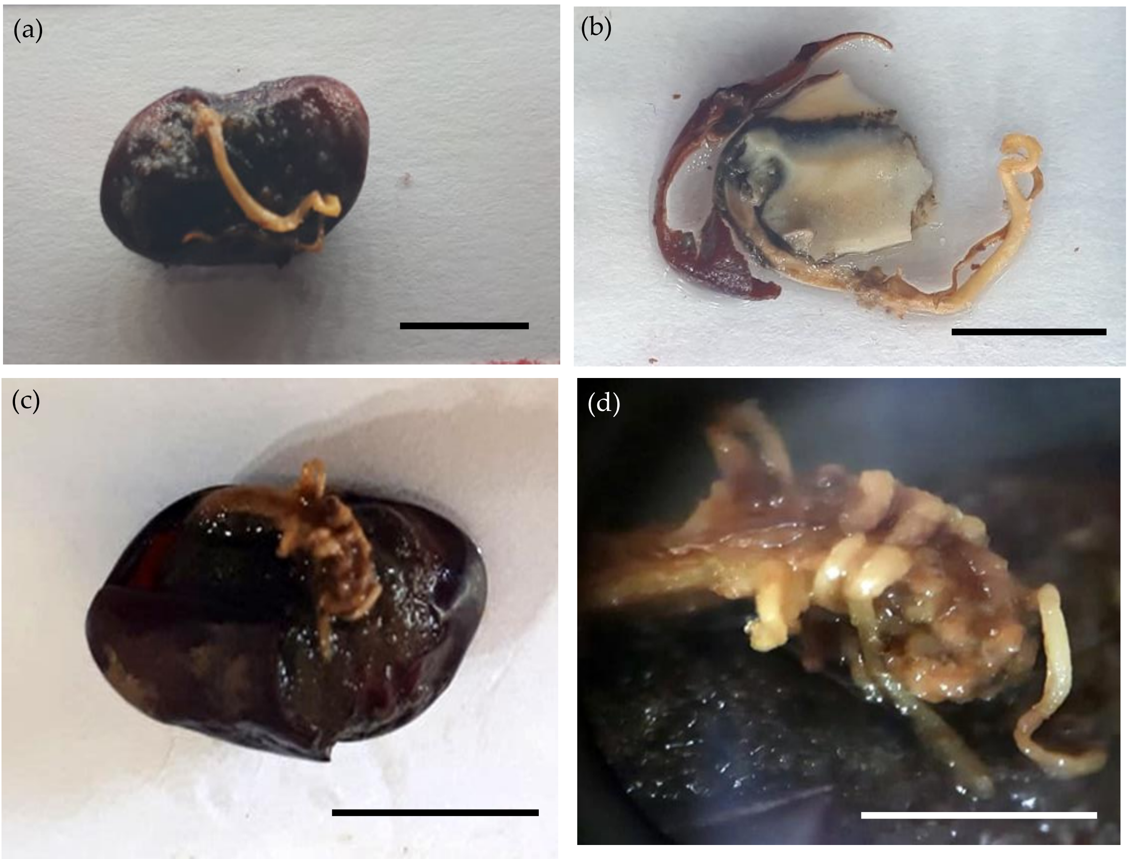

Figure S1. Representative seeds that were treated with [D-Leu¹]MC-LR (**a**, **b**) or MC-LR (**c**, **d**) and that started the germination process but failed to develop a seedling. Note the atrophied primary root, secondary root emerging from the hypocotyl (**c**, **d**) and necrotic areas in cotyledon (**b**). Scales lines: 1 cm (black), 5 mm (white).

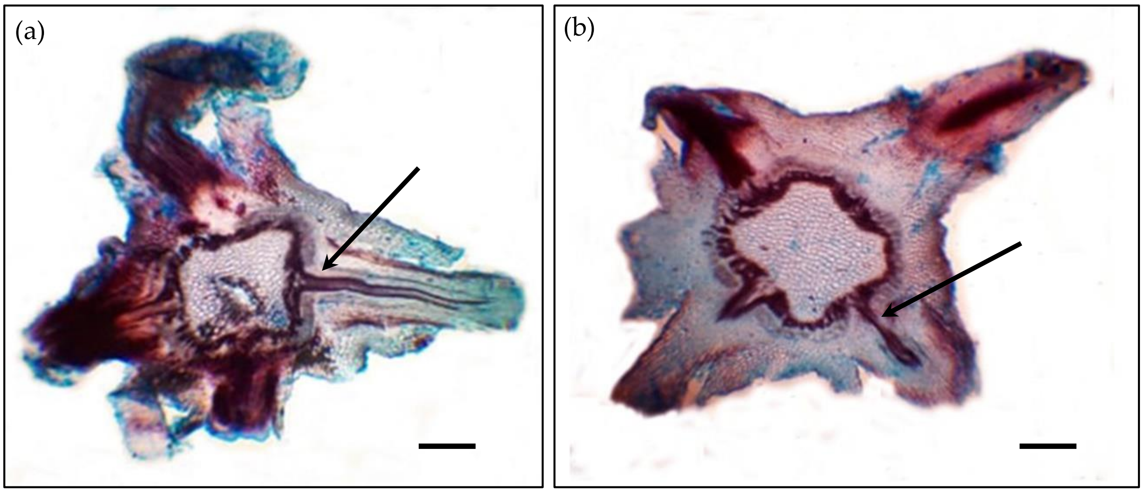

Figure S2. Representative histological sections stained with Alcian Blue/Safranin (40×) from roots of seeds treated with [D-Leu[1]]MC-LR (**a**) or MC-LR (**b**) that started germination but failed in seedling development. Note that numerous secondary roots emerge from the hypocotyl central vascular cylinder (black arrows). Scales lines 100 µm.

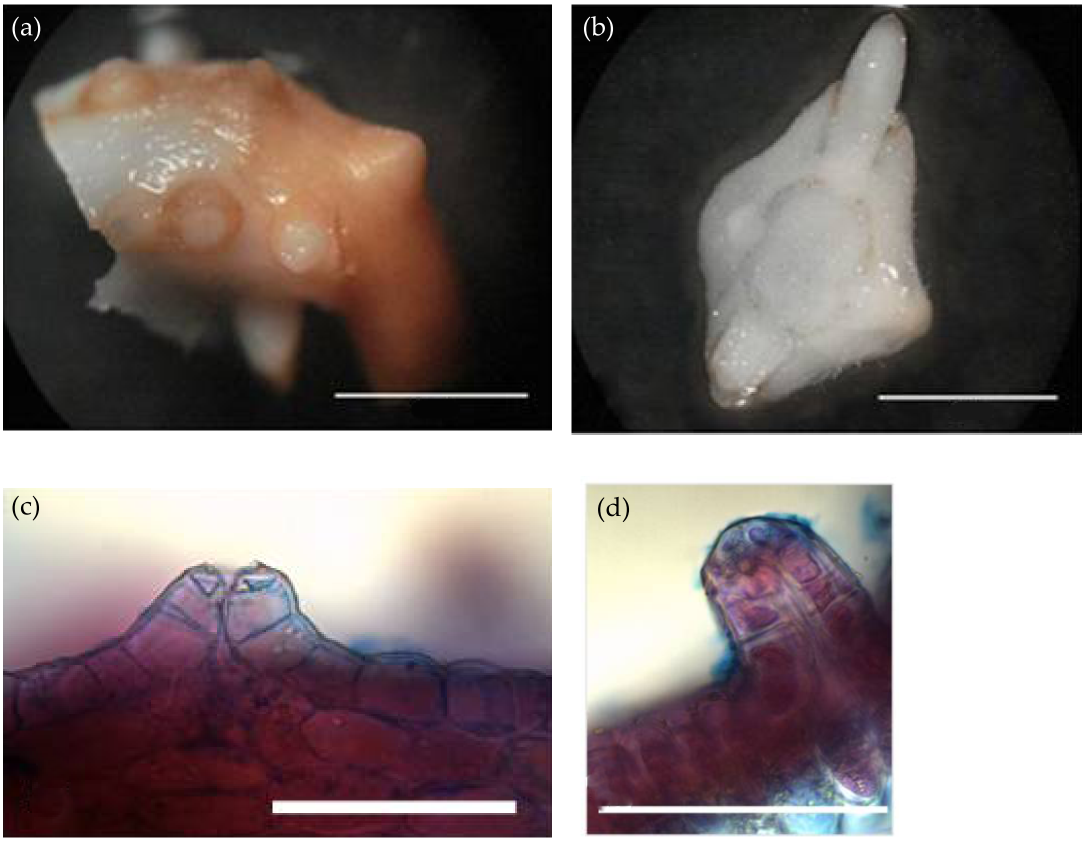

Figure S3. Representative hypocotyl surface view (**a**, **b**) and Alcian Blue/Safranin stained cross section (**c**, **d**) (100×) from MC-LR-treated seeds that started germinating but failed to develop seedlings. Note the presence of roots in the hypocotyl (**a**, **b**) and the presence of raised or "in column" stomata (**c**, **d**). These alterations were observed only for MC-LR, indicating the attempt to develop seedlings under the MC-LR exposure condition. The lack of attempts to develop seedlings in the [D-Leu[1]]MC-LR treatment is in line with higher toxic potency of this toxin with respect to MC-LR. Scales lines: 2mm (**a**, **b**); 100 µm (**c**, **d**).
